# Common and unique features of glycosylation and glycosyltransferases in African trypanosomes

**DOI:** 10.1042/BCJ20210778

**Published:** 2022-09-06

**Authors:** Samuel M. Duncan, Michael A.J. Ferguson

**Affiliations:** Wellcome Centre for Anti-Infectives Research, School of Life Sciences, University of Dundee, Dundee DD1 5HN, U.K.

**Keywords:** evolutionary biology, glycosylation, glycosyltransferases, trypanosomes

## Abstract

Eukaryotic protein glycosylation is mediated by glycosyl- and oligosaccharyl-transferases. Here, we describe how African trypanosomes exhibit both evolutionary conservation and significant divergence compared with other eukaryotes in how they synthesise their glycoproteins. The kinetoplastid parasites have conserved components of the dolichol-cycle and oligosaccharyltransferases (OSTs) of protein *N*-glycosylation, and of glycosylphosphatidylinositol (GPI) anchor biosynthesis and transfer to protein. However, some components are missing, and they process and decorate their *N*-glycans and GPI anchors in unique ways. To do so, they appear to have evolved a distinct and functionally flexible glycosyltransferases (GT) family, the GT67 family, from an ancestral eukaryotic β3GT gene. The expansion and/or loss of GT67 genes appears to be dependent on parasite biology. Some appear to correlate with the obligate passage of parasites through an insect vector, suggesting they were acquired through GT67 gene expansion to assist insect vector (tsetse fly) colonisation. Others appear to have been lost in species that subsequently adopted contaminative transmission. We also highlight the recent discovery of a novel and essential GT11 family of kinetoplastid parasite fucosyltransferases that are uniquely localised to the mitochondria of *Trypanosoma brucei* and *Leishmania major*. The origins of these kinetoplastid *FUT1* genes, and additional putative mitochondrial *GT* genes, are discussed.

## Introduction

The protozoan parasite *Trypanosoma brucei* cause African animal trypanosomiasis (or nagana) in cattle and human African trypanosomiasis (HAT) in humans. These diseases are generally fatal if not treated and the available therapeutics, while improving for HAT, are far from optimal. Currently, with tsetse fly control and test-and-treat surveillance, reported cases of HAT in sub-Saharan Africa are, thankfully, low. However, the disease burden in cattle significantly affects economic output and agricultural productivity [1].

Many African trypanosome species, for example, *T. brucei* spp., *T. congolense* and *T. suis*, have complex life cycles that involve obligate differentiation events between proliferative (colonising) and non-proliferating (transmissible) stages to occupy and pass between their mammalian hosts and tsetse fly vectors. Others have lost the ability to infect tsetse vectors and transmit by either venereal (*T. equiperdum*) or contaminative routes via haematophagous fly bites (*T. evansi*); *T. vivax*, which has a limited lifecycle in tsetse, is also transmitted primarily by venereal and hematophagous fly bite routes.

**Figure 1.**
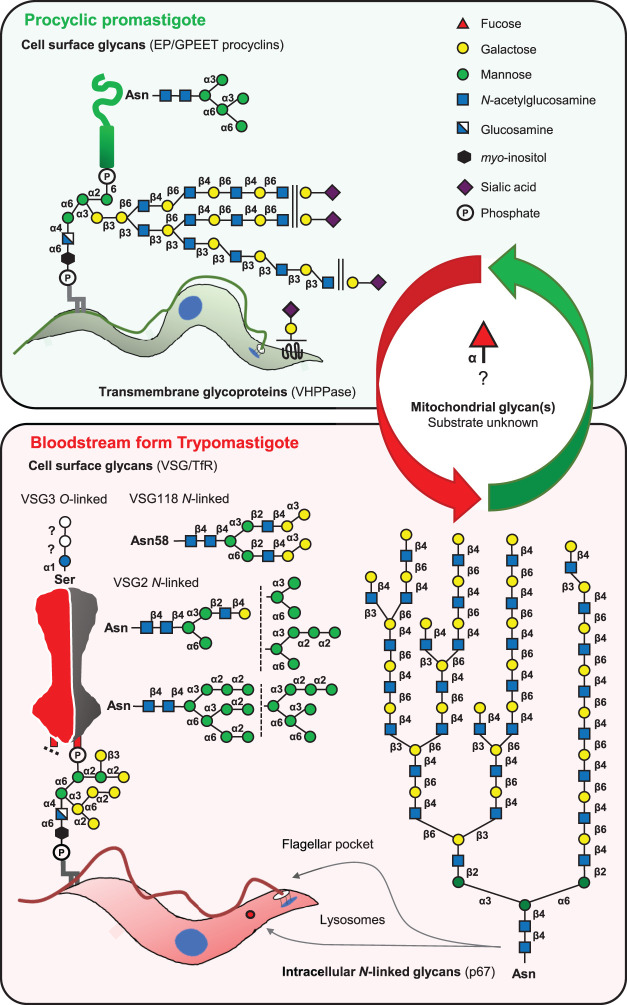
Summary of the glycan and GT repertoires of *T. brucei*. The tsetse midgut-dwelling procyclic form (PCF) cells express GPI-anchored and *N*-glycosylated procyclin glycoproteins with simple Man_5_GlcNAc_2_ oligomannose *N*-glycans and GPI anchors with extensively modified GPI-anchor glycans. The mammalian host-dwelling bloodstream form (BSF) cells express variant surface glycoproteins (VSGs) that can contain oligomannose (triantennary Man_9_GlcNAc_2_ to Man_5_GlcNAc_2_), paucimannose (biantennary Man_5_GlcNAc_2_ to Man_3_GlcNAc_2_) and small complex *N*-glycans. Some VSGs are also *O*-glycosylated, as indicated. In addition, other flagellar pocket and endosomal/lysosomal glycoproteins, such as p67, bear giant poly-LacNAc-containing *N*-glycans in the BSF lifecycle stage. In contrast, the BSF GPI-anchor sidechains are smaller than those of PCF cells, containing up to 6 Gal residues.

The bloodstream form (BSF) trypomastigote and procyclic form (PCF) promastigote lifecycle stages that occupy mammalian host and insect vector niches, respectively, exhibit dramatic changes in cellular metabolism, morphology and cell surface molecular architecture. The latter is illustrated for *T. brucei* in (Figure 1).

Eukaryotic protein *N*-glycosylation involves the transfer of an oligosaccharide from a lipid-linked oligosaccharide (LLO), made by the dolichol-cycle, to NXS/T acceptor sequons in proteins sequestered into the endoplasmic reticulum (ER). This transfer is mediated by an oligosaccharyltransferase (OST) [2,3]. GPI membrane anchors are also pre-assembled in the ER and transferred *en bloc* via GPI-transamidase in the lumen of the ER to a subset of proteins bearing a C-terminal peptide extension [4]. The core *N*-glycan and GPI structures are then processed in the ER and in the Golgi apparatus to mature structures. Most of the genes encoding GTs involved in the dolichol-cycle (*ALG* genes) and GPI precursor biosynthesis (*GPI* genes), which belong to the GT-C fold class [5], have been found in the *T. brucei* genome by predicted amino acid sequence homology [6]. In a few cases, the functionalities of *ALG* and *GPI* GT genes have been experimentally confirmed [7–10]. However, the dolichol-cycle glucosyltransferases *ALG6*, *ALG8* and *ALG10* are notably missing, as are homologues of GTs that elaborate *N*-glycans and GPI-side chains in other organisms. This is despite many of the processed parasite *N*-glycan structures being identical with those of higher eukaryotes. Thus, for example, one cannot easily find orthologues of the genes GnTI and GnTII (which add GlcNAc to the 3- and 6-branches of the conserved Man_3_GlcNAc_2_ core and thus initiate the formation of complex *N*-glycans) or of B4GalT that makes Galβ1–4GlcNAc (LacNAc) structures.

Such processing GTs are generally type-2 membrane proteins localised to the Golgi apparatus and are defined by their nucleotide sugar donor, the structure of their aglycone acceptor, the anomericity of the transferred sugar (α or β) and the inter-sugar glycosidic linkage (e.g. 1–2, 1–3, 1–4 or 1–6). For example, a UDP-Gal : βGlcNAc β1–4 Gal-transferase makes Galβ1–4GlcNAcβ1-*O*-R from a UDP-Gal donor and a GlcNAcβ1-*O*-R acceptor. The genes encoding GTs often exhibit significant expansion through evolutionary pressure to catalyse a repertoire of related glycosidic linkages [11]. Most GTs are classified based on their 3D fold topology, where GT-As have two closely adjoining β/α/β Rossmann domains whilst GT-Bs consist of two facing β/α/β Rossmann domains that are linked flexibly [11]. Overall fold classification is not a predictor of the catalytic mechanism, as both inverting (β) and retaining (α) GTs have been characterised with GT-A and GT-B topologies. Phylogenetic analyses of GT-A fold GTs indicate that inverting and retaining mechanisms emerged independently many times during evolution [12]. The GTs are further categorised into distinct GT families in the carbohydrate enzyme (CAZy) database, based on protein sequence and structural similarities [13]. Experimental data on GT family members with respect to their inverting or retaining transfer mechanisms and nucleotide sugar specificities allows conservative predictions on the specificities of unstudied fellow GT family members. The recent application of GT sequence deep mining and machine learning approaches are showing promise in predicting the mechanistic function of GTs based on alterations to their common core [14]. Nevertheless, mechanistic predictions from primary protein sequence remain tentative.

The CAZy database contains over a hundred sequence-based GT families and many or all families are encoded by most organisms. However, this is not the case in *T. brucei* where several common GT families are missing and where a particular GT family (GT67), unique to kinetoplastids, has emerged and expanded. There are twenty of these genes in the *T. brucei* genome and, so far, the functions of five of them have been studied [15–20].

In this review we summarise our current knowledge of protein glycosylation in *T. brucei*, discuss the kinetoplastid-specific GT67 family, and highlight recent discoveries on a novel kintepolastid-specific mitochondrial fucosyltransferase (FUT) and other putative mitochondrial GTs.

## Protein glycosylation in *T. brucei*

### Glycan structures in BSF *T. brucei*

In the mammalian host, the proliferative BSFs reside within the bloodstream, lymphatics and sub-cutaneous and adipose tissue niches [21,22]. BSF trypanosomes survive by expressing around 5 million variant surface glycoprotein (VSG) homodimers tethered to the cell surface via GPI anchors [23,24]. The VSGs produce a dense, yet mobile [23], proteinaceous coat that protects the plasma membrane from components of the innate immune response, such as complement, whilst enabling the diffusion of small nutrient molecules for uptake into the cell via transmembrane transporters [23,25,26]. These VSG molecules are immunogenic and the parasite survives the adaptive immune response by antigenic variation, whereby parasites express alternative VSGs from a large repertoire of genes [27]. VSGs are classified on amino acid sequence motifs, and these sub-types generally share glycosylation features, such the attachment of one, two or three *N*-linked oligomannose, paucimannose or complex *N*-glycans and GPI-anchor sidechains of between zero to five α-linked Gal residues and zero or one β-linked Gal residue (Figure 1) [25,26,28]. The *N*-glycosylation of VSG is an important modification which insulates the protein core from intermolecular interactions with adjacent surface proteins, enabling dense packing to occur at a level approaching the molecular crowding threshold [29]. Additionally, *O*-glycosylation of certain VSGs has been identified as a further mechanism by which African trypanosomes generate additional antigenic variation. Here, a serine residue at the top of the VSG molecule bears an αGlc residue that can be further modified by 1 or 2 hexose residues to generate heterogeneity that delays the onset of a sterilising host immune response [30].

There are several other less-abundant glycoproteins expressed by BSF *T. brucei*, but only the flagellar pocket VSG-like transferrin receptor (TfR) and the lysosomal/endosomal p67 hydrolase have been analysed in any detail for carbohydrate structure. The TfR contains a VSG-type GPI anchor [31], albeit on only one of its two subunits, and both TfR and BSF p67 contain oligomannose, paucimannose [32] and poly-*N*-acetyllactosamine, i.e. poly-Galβ1–4GlcNAc (poly-LacNAc), containing complex *N*-glycans [33]. The latter include the largest neutral *N*-glycan structures yet described in eukaryotes [34]. Thus, in contrast with their relatively short GPI sidechain glycans, BSF *T. brucei* can express extremely large complex *N*-glycans. The poly-LacNAc *N*-glycans have been suggested to play a role in endocytosis [35], but their exact function is unknown.

### Glycan structures in PCF *T. brucei*

The PCF cell surface contains a partially characterised high-molecular mass glycoconjugate [36], abundant GPI-anchored glycoproteins called procyclins [37] and free GPI glycolipids [38,39]. The procyclins are composed of rod-like polyanionic dipeptide (EP) or pentapeptide (GPEET) repeats with or without a single triantennary Man_5_GlcNAc_2_ *N*-linked oligomannose glycan [40] and without or with threonine phosphorylation [41], respectively. Both types of procyclin share the largest and most complex GPI-anchor sidechains characterised to date. These glycans are composed of branched poly-LacNAc and poly-lacto-*N*-biose (LNB; Galβ1–3GlcNAc) containing structures terminating in βGal [40,42] that can be further modified by α2–3-linked sialic acid residues by the action of cell surface GPI-anchored trans-sialidase [43]. Surface sialylation with host blood meal-derived sialic acids plays a role in efficient tsetse fly colonisation [44] whilst the rod-like procyclins are thought to shield susceptible surface proteins from proteolytic attack in the tsetse midgut [45]. Therefore, in contrast with the densely packed, proteinaceous VSG coat of BSF cells, the PCF cells express a surface glycocalyx composed of elaborate GPI sidechain glycans overlayed by polyanionic peptidic rods and interlaced with high-molecular mass glycoconjugates. Significantly, while wild-type PCF parasites express extremely complex GPI sidechains, they only express simple oligomannose *N*-glycans [28,40] (Figure 1).

Of note, the tsetse midgut PCF of *T. congolense* expresses a different family of glycoproteins, called glutamic acid and alanine-rich glycoproteins (GARPs), in place of procyclins. The GARPs are also GPI-anchored molecules, but with small GPI sidechains, no *N*-linked glycans and Gal and Man containing glycans linked through phosphate to Thr residues [46].

### Glycan structures in other lifecycle stages

In *T. brucei*, the other lifecycle stages are less accessible than BSF and PCF but *T. brucei* epimastigote forms are known to express a GPI-anchored alanine-rich protein called BARP [47,48] and metacyclic trypomastigote forms express a related GPI-anchored metacyclic invariant surface protein (MISP) [49]. However, as for most BSF and PCF glycoproteins, there are no structural data on their GPI sidechains and/or *N*-linked glycans.

## Conserved and divergent aspects of protein *N*-glycosylation and protein quality control in *T. brucei*

Eukaryotic OSTs are generally hetero-oligomeric complexes where the catalytic-subunit (STT3) is associated with seven or eight additional subunits. These OSTs generally transfer Glc_3_Man_9_GlcNAc_2_ from the mature LLO of the dolichol-cycle to NXS/T sequons in the lumen of the ER [2,3,50]. The transferred glycans then undergo processing by ER glucosidase I and II and ER α-mannosidase I to generate oligomannose structures. When these arrive in the Golgi apparatus, they can be further processed to complex and hybrid structures through the action of Golgi α-mannosidase II and a variety of GTs [3,51].

In *T. brucei* this canonical pattern of protein *N*-glycosylation is modified, as first noted by Bangs, Englund and colleagues who observed that some *N*-glycosylation sites in *T. brucei* VSGs are occupied by endoglycosidase-H resistant N-glycans immediately after VSG synthesis in the ER [52]. The anomalies of protein *N*-glycosylation in *T. brucei* are: Firstly, its OST activity is provided by STT3 gene products alone [53]. Secondly, the largest LLO made by the parasite is Man_9_GlcNAc_2_ [8,54]. Thirdly, its three STT3 genes encode OSTs with significantly different donor and acceptor specificities [8,10,53,55,56]. The consequence of this is a radically different *N*-glycosylation system, whereby *N*-glycosylation efficiency is very high and the type of mature glycans on specific *N*-glycosylation sites is primarily controlled by the net charge around the glycosylation site. Thus, TbSTT3A prefers sequons in an acidic environment and specifically transfers biantennary Man_5_GlcNAc_2_ from the LLO Man_5_GlcNAc_2_-PP-dolichol, and TbSTT3B acts on all remaining sequons and specifically transfers triantennary Man_9_GlcNAc_2_ from the LLO Man_9_GlcNAc_2_-PP-dolichol. Since the organism does not contain a Golgi α-mannosidase II activity, the consequence is that only TbSTT3A acidic sequon sites are destined to be processed to paucimannose and/or complex *N*-glycans whereas TbSTT3B sequon sites are destined to contain only oligomannose structures. Interestingly, neither TbSTT3A nor TbSTT3B appears to be essential for BSF parasites *in vitro*, but both are essential *in vivo* [56]. The role of TbSTT3C is unclear as it has not been detected at the protein level in BSF or PCF cells. However, its effects on protein glycosylation when transferred to yeast suggest it has a hybrid specificity, preferring acidic sequons like TbSTT3A but transferring Man_9_GlcNAc_2_ like TbSTT3B [57].

The preponderance of oligomannose *N*-glycans in PCF glycoproteins, versus both oligomannose and paucimannose/complex *N*-glycans in BSF glycoproteins, is easily understood when the expression of TbSTT3A and TbSTT3B are compared at the protein level (Figure 3). Thus, TbSTT3B is highly expressed in PCF and BSF, whereas TbSTT3A is only highly expressed in BSF cells.

Since the *T. brucei* LLOs do not contain glucose, the parasites do not have an ER glucosidase I. However, they do have UDP-Glc: glycoprotein glucosyltransferase (UGGT), ER glucosidase II and calreticulin so that newly synthesised glycoproteins in the ER can undergo quality control cycles of *N*-glycan α-glucosylation (via UGGT), attempts at protein folding via calreticulin and its associated oxidoreductases, and de-glucosylation (via ER glucosidase II). The *T. brucei* UGGT shares a typical GT24 family domain at its C-terminus [58] but the activity of TbUGGT has diverged from a strict specificity for Man_9_GlcNac_2_ to a broad specificity for any *N*-glycan structure containing an intact A-branch [59]. In common with other organisms, TbUGGT plays a role in protection from heat shock, such that BSF TbUGGT *null* mutants cannot tolerate a 37–40°C temperature shift [59]. However, unlike other organism such as *Schizosaccharomyces pombe*, the *TbUGGT null* mutant does not up-regulate chaperones such as Grp78 or BiP upon heat shock or following tunicamycin treatment. It appears, therefore, that the accumulation of unfolded proteins upon ER stress is not sensed in BSF *T. brucei*, a conclusion also reached by Tiengwe *et al*. [60]. This ‘chaperones always on' condition is likely a consequence of the extremely high glycoprotein flux required to export VSG in BSF *T. brucei* to form a dense surface coat. Consistent with this proposal of a *T. brucei*-specific adaptation, the *T. cruzi* UGGT *null* mutant does up-regulate ER chaperones Grp78 and BiP [61].

An interesting phenomenon is that both BSF and PCF *T. brucei* show plasticity in protein *N*-glycosylation when challenged with toxic lectins or other carbohydrate-binding agents. For example, PCF cells express small hybrid *N*-glycans in place of oligomannose glycans when challenged with Concanavalin-A [54] and BSF cells alter the expression of their TbSTT3 genes and create TbSTT3 chimeric genes when challenged with lectins and other agents [62–64].

## Divergent and convergent evolution of GT67 family GTs

As mentioned previously, despite conserved glycan structural motifs between *T. brucei* and other eukaryotes one cannot find orthologues of the genes GnTI and GnTII (which add GlcNAc to the 3- and 6-branches of the conserved Man_3_GlcNAc_2_ core and thus initiate the formation of complex *N*-glycans), or of B3GnTI that makes GlcNacβ1–3Gal structures, or β3GalTI that makes Galβ1–3GlcNAc structures, or GCNT2 that makes GlcNacβ1–6Gal structures. Each of these belong to distinct CAZy GT families. Instead, the genes encoding these five activities belong to the kinetoplastid-unique GT67 family [13]. This GT-A fold family has three motifs that are very similar to the (I/L)RXXWG, (F/Y)(V/L/M)XXX-DXD, (ED)D(A/V)(Y/F)XGX(C/S) motifs conserved among members of the mammalian β3GT superfamily [16]. The comparable motifs in the *T. brucei* genes are WG, Y(I,V,F)XKXDDD, and ED(A/V/I/L/M)(M/L)X(G/A). The GT67 family, therefore, appears to have diverged from the normal eukaryotic β3GT lineages and then expanded and evolved such that GT67 GTs can take the place of GT13 [17], GT14 [16], GT16 [18], GT31 [19] and GT49 [15] (and probably more) GT family members.

Phylogenetic analysis of the GT67 gene family reveals that it separates into two distinct clades, one for the *Leishmania* (not discussed here but reviewed in [65]) and one for the trypanosomatids [66], indicative of the disparate evolutionary pressures exerted on these parasite groups.

Fifteen of the twenty *T. brucei* GT67 gene products remain uncharacterised, and specificity predictions based on sequence analyses alone are of limited value. In contrast, the elegant interspecies comparisons of GT67 family members amongst trypanosomatids performed by Pereira and Jackson, referred to by them as UDP-dependent GTs (UGTs) [66], provides some useful clues to TbGT function. Thus, we might assume that GT genes in *T. brucei* that are shared with *T. vivax* are more likely to encode those required for the synthesis of BSF structures, whereas those not shared with *T. vivax* (which does not have an obligate transmission through the tsetse fly) are more likely to encode those required for the synthesis of PCF structures.

We should note that while these inferred likelihoods of GT requirements in BSF and PCF lifecycle stages are useful guides, they do not preclude predominantly BSF- or PCF-expressed GT activities appearing in the other lifecycle stage. For example, under selective pressure from the lectin Concanavalin-A [54] or mutagenesis of the *TbALG12* gene [7], PCF cells stop making oligomannose *N*-glycans and the action TbGnTI or TbGnTII on the paucimannose structures that replace them can be detected. Conversely, similar GPI-anchor sidechains to those found in abundance on PCF procyclins can be found on certain substrates, like the ESAG6 subunit of the transferrin receptor, in BSF cells when steric constraints around the GPI anchor are relaxed [33].

**Figure 2.**
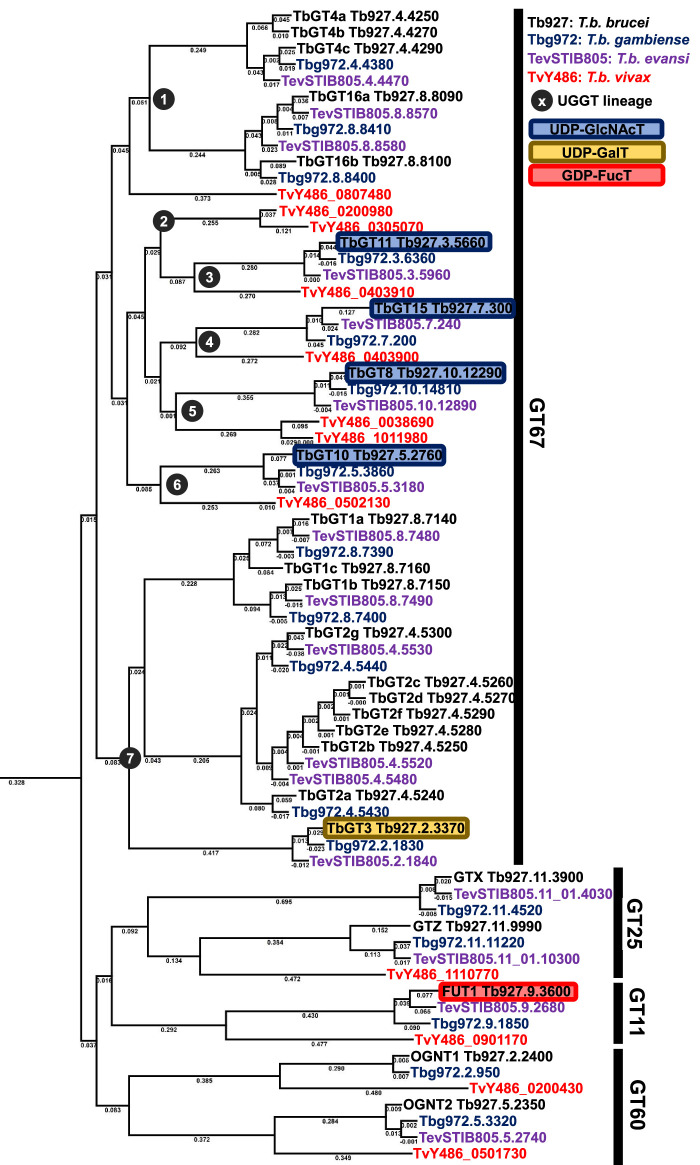
*GT* repertoire of *T. brucei*. Phylogenetic analysis of *T. b. brucei*, *T. b. gambiense*, *T. evansi* and *T. vivax* GT genes. The lineages within the GT67 family are according to [66] and the *T. brucei* GT sub-families within those lineages (e.g. TbGT1 to TbGT15) are according to [15,18]. Those TbGTs that appear in proteomics data are shown in Figure 3.

**Figure 3.**
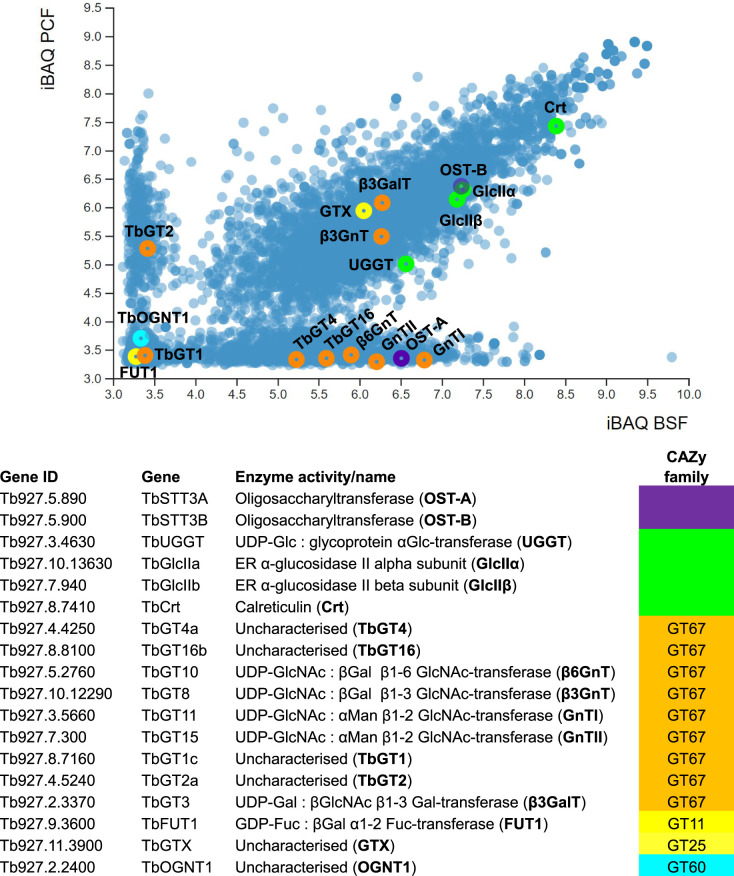
Protein abundance data for enzymes and proteins involved in protein glycosylation in *T. brucei*. The subset of gene products detected by proteomics associated with oligosaccharide transfer (purple), ER quality control (green) together with the *T. brucei* GT67 glycosyltransferases (orange), GT11 and GT25 mitochondrial glycosyltransferases (yellow) and GT60 glycosyltransferase (cyan) are shown. Gene IDs, gene names and encoded activities (and abbreviated names) are shown in the table. The latter are plotted according to quantitative proteomics values (iBAQ values) in the BSF (*x*-axis) and PCF (*y*-axis) total cell proteomes [68] using the tool described in [69].

The phylogenetic analysis of Pereira and Jackson revealed seven distinct trypanosomatid GT gene lineages within the GT67 family. Here, we have performed similar analysis and included *T. evansi* [67], which does not differentiate to the PCF stage due to the absence of a mitochondrial genome, and *T. gambiense*, the human pathogenic strain closely related to *T. b. brucei* (Figure 2). We overlay and interpret this phylogenetic analysis with the available data on *T. brucei* GT67 functions [15–19] and on TbGT, TbOST and glycan-processing enzyme protein expression data from quantitative proteomics [68]. The latter expressed graphically in (Figure 3) using the tools in described in [69].

### Lineage 1

There is a single gene of this lineage in *T. vivax* which seems to have undergone expansion in *T. b. brucei* to generate the *TbGT4* (three copies) and *TbGT16* (two copies) gene sub-families. These sub-families are also present in *T. b. gambiense* and *T. evansi*. The greater expression at the protein level of this lineage in BSF compared with PCF cells (Figure 3) suggests they may play a BSF-specific function. Given the notable absence of UDP-Gal : βGlcNAc β1–4 Gal-transferases needed to synthesise the abundant *N*-linked poly-LacNAc structures in BSF *T. brucei* [34], we postulate that one or more of these lineage 1 genes may encode GTs with GT7 β4GalTI-like activity.

### Lineage 2

This is a *T. vivax*-specific gene family and structural data on *T. vivax* glycans is scant [70], making inferences about the activity of the GTs they encode difficult. Furthermore, sequence analysis indicates they may be fragment rather than functional sequences. We postulate that these gene products, if functional, may encode activities similar to their closest relatives in lineage 3.

### Lineages 3 and 4

Lineage 3 contains the gene encoding TbGT11, which has been experimentally shown to be in the Golgi and to perform the same function as GT13 family GnTI in other eukaryotes [17]. In other words, it adds βGlcNAc in 1–2 linkage to the 3-arm of the conserved Man_3_GlcNAc_2_ core. However, TbGnTI does so with unique acceptor specificity: Whereas canonical GT13 GnTIs work on Man_5_GlcNAc_2_, which is then processed to GlcNAc_1_Man_3_GlcNAc_2_ by Golgi α-mannosidase II (the latter is absent from *T. brucei*), GT67 TbGnTI works directly on Man_3_GlcNAc_2_.

Lineage 4 contains the gene encoding TbGT15, a Golgi enzyme that performs the same function as GT16 family GnTII, adding βGlcNAc in 1–2 linkage to the 6-arm of the conserved Man_3_GlcNAc_2_ core [16]. However, whereas canonical GT16 GnTIIs work on GlcNAc_1_Man_3_GlcNAc_2_, GT67 TbGnTII can operate with nothing or anything substituting the 3-arm αMan residue. This also means that TbGnTI and TbGnTII can operate independently of each other, unlike the strict GnTI followed by Golgi α-mannosidase II followed by GnTII sequence in other eukaryotes [71].

In mice, homozygous *null* mutants of *GnTI* (*Mgat1^−/−^*) or *GnTII* (*Mgat2^−/−^*) do not survive beyond embryonic day 10 or postnatal week 1, respectively [72,73]. In contrast, *T. brucei* BSF *TbGnTI* and *TbGnTII null* mutants both survive in culture and in mice. However, the absence of either leads to compensation by extension and elaboration of the opposing arm. Thus, deletion of *TbGnTI* increases the decoration of the 6-arm [17] and deletion of *TbGnTII* increases the decoration of the 3-arm [16]. Attempts to inhibit the formation of complex *N*-glycans entirely in BSF *T. brucei* by generating a double *null* mutant for *TbGnTI* and *TbGnTII* have been unsuccessful (unpublished data), suggesting that complex *N*-glycans *per se* are likely to be essential in BSF *T. brucei*. Consistent with this, we were also unable to make a BSF *TbSTT3A null* mutant [74], even though we could knock down TbSTT3A substantially by RNAi in a heterozygote [56]. These data also suggest that some expression of TbSTT3A, and therefore at least some capacity to make complex and/or paucimannose *N*-glycans, is essential.

Finally, lineage the 3 and 4 TbGnTI and TbGnTII enzymes are expressed predominantly in BSF cells, consistent with their requirement for TbSTT3A co-expression to make complex *N*-glycans (Figure 3).

### Lineages 5 and 6

All four trypanosome species contain one or two (*T. vivax*) copies of lineage 5 and 6 GTs. Lineage 5 contains the gene encoding TbGT8, which performs the same function as GT49 family B3GnTI in other eukaryotes [15]. It adds βGlcNAc in 1–3 linkage to βGal residues. Lineage 6 contains the gene encoding TbGT10, which performs the same function as GT14 family GCNT, adding βGlcNAc in 1–6 linkage to βGal residues [18]. TbGT8 and TbGT10 are active in both BSF and PCF cells. Together, they synthesise -4GlcNAcβ1–6(-4GlcNAcβ1–3)Galβ1-branch points in BSF complex *N*-glycans and in PCF GPI sidechains. Independently, they extend linear chains of poly-LacNAc repeats in BSF complex *N*-glycans (both conventional Galβ1–4GlcNAcβ1–3Galβ1–4GlcNAc repeats via TbGT8 and the less common Galβ1–4GlcNAcβ1–6Galβ1–4GlcNAc repeats that predominate in BSF *T. brucei* via TbGT10), and the Galβ1–4GlcNAcβ1–6Galβ1–4GlcNAc poly-LacNAc (via TbGT10) and Galβ1–3GlcNAcβ1–3Galβ1–3GlcNAc LNB repeats (via TbGT8) in PCF GPI sidechains. Interestingly, while TbGT8 and TbGT10 both operate in BSF and PCF cells, and although TbGT8 protein levels are similar in BSF and PCF cells, TbGT10 protein levels are significantly lower in PCF cells despite its role in GPI anchor elaboration (Figure 3).

There is interplay between TbGT8 and TbGT10 in that elimination of either not only reduces -4GlcNAcβ1–6(-4GlcNAcβ1–3)Galβ1-branch points but also elicits compensatory linear glycosylation by the other [17], reminiscent of the compensatory redundancy between TbGnTI and TbGnTII described above.

Analysis of the BSF *TbGT10 null* mutant [18] showed that impairment of Galβ1–4GlcNAcβ1–6Galβ1–4GlcNAc poly-LacNAc synthesis also perturbs the proteolytic processing of the essential [75] lysosomal/endosomal glycoprotein p67. While this had a minor growth phenotype in culture, these parasites were still infectious to mice. Thus, there does not appear to be any crucial role for Galβ1–4GlcNAcβ1–6Galβ1–4GlcNAc poly-LacNAc synthesis, or indeed for wild-type p67 processing, in BSF *T. brucei*.

### Lineage 7

Lineage 7 has undergone significant expansion in the genomes of *T. brucei*, *T. b. gambiense* and *T. evansi* but it is absent from *T. vivax*. A single lineage 7 member (TbGT3) has been analysed to date and shown to be expressed in both BSF and PCF cells (Figure 3). In BSF cells it plays some (undefined) role in glycoprotein processing, as judged by wheat germ agglutinin (WGA) lectin blotting [19]. However, it is well characterised in PCF *T. brucei* as a UDP-Gal : βGlcNAc-GPI β1–3 Gal-transferase elaborating GPI-anchor sidechains [19]. TbGT3 is, therefore, functionally related to GT31 family B3GALT1 that also makes LNB (Galβ1–3GlcNAc) structures.

The absence of lineage 7 in *T. vivax*, the defined function of TbGT3 in PCF cells and the higher protein expression of the lineage 7 TbGT2 sub-family (encoded by seven closely related genes) in PCF cells (Figure 3), suggest that at least some lineage 7 GTs may be primarily involved in the decoration of the PCF stage GPI-anchor sidechains. We further postulate that lineage 7 PCF expression-specific TbGT2 sub-family genes may encode the elusive UDP-Gal : βGlcNAc-GPI β1–4 Gal-transferases required for the -6Galβ1–4GlcNAcβ1-poly-LacNAc chains found in PCF GPI-anchor sidechains.

The presence of lineage 7 genes in *T. evansi*, which does not have a PCF stage, runs counter to this argument but may be a function of the phylogenetic proximity of *T. evansi* to *T. brucei,* such that lineage 7 GTs may yet to be lost from the *T. evensi* genome, and/or it may reflect some need for lineage 7 GTs in *T. evansi* (but not *T. vivax*), BSF cells.

The lineage 7 TbGT1 sub-family has been detected by proteomics in both BSF and PCF cells [68], but the absolute expression levels are very low (Figure 3), and their specificities and functions are unknown.

Taken together, these data suggest the evolution of a large trypanosome GT67 gene family to generate the UDP-GlcNAc/UDP-Gal glycosyltransferase repertoire necessary for the biosynthesis of substantial parts of their uniquely complex *N*-linked and GPI-anchor side chain glycans. This serves as is a prime example of convergent evolution, whereby the GT67 enzymes exhibit functional relatedness with several families of metazoan GTs yet derive from an ancestrally distinct β3GT. Our assessments of lineages 3, 4, 5 and 6 indicate that they encode BSF-specific trypanosomatid GTs necessary for complex *N*-linked glycan synthesis in BSF cells. Furthermore, we propose that life cycle stage-dependent β1–4 Gal-transferases are encoded by lineages 1 (in BSF cells) and 7 (in PCF cells). Reverse genetics experiments are required to confirm this hypothesis.

## Mitochondrial glycosyltransferases of kinetoplastea

### GT11 family FUT1

The discovery that the biosynthesis of the nucleotide sugar GDP-fucose was required for parasite growth in *T. brucei* [76] and in *Leishmania major* [77], suggested that these organisms contain one or more essential FUT genes. A single GT11 family FUT, *TbFUT1*, was identified in the *T. brucei* genome and shown to be essential in BSF and PCF cells [78]. Similarly, the *L. major* orthologue (*LmjFUT1*) is essential for cell growth [79]. The most curious feature of TbFUT1 and LmjFUT1 is their localisation to the parasite mitochondria [78,79]. Recombinant TbFUT1 has GDP-Fuc : βGal α1–2-fucosyltransferase activity, common amongst GT11 family enzymes, with an apparent preference for acceptor substrates containing a terminal LNB (Galβ1–3GlcNAc) motif [78]. The origin of FUT1 is unlike that of the GT67 family which expanded from a common eukaryotic β3GT ancestor. Instead, phylogenetic analysis of *FUT1* genes in kinetoplastids indicates it was inherited from a bacterial *FUT1* by horizontal gene transfer via a nucleocytoplasmic large DNA virus [80]. The fact that *FUT1* is both mitochondrial and essential in both organisms studied to date, and displays highly conserved sequence similarity and genomic synteny across the kinetoplastea, suggests that the αFucT activity it encodes may play a common and crucial role in mitochondrial function in this group of organisms.

Exactly what role(s) mitochondrial fucosylation might play in kinetoplastids remains to be determined. Whereas recombinant TbFUT1 and LmjFUT1 have been shown to fucosylate exogenous peptide and/or glycan substrates, their native mitochondrial substrates have not yet been identified. Phenotypic analysis of a TbFUT1 conditional *null* mutant in BSF cells reveals that depleting TbFUT1 causes a loss of mitochondrial membrane potential, linked to the activity of the F_o_F_1_-ATP synthase [78]. BSF *T. brucei* lack the proton pumping respiratory chain complexes normally expressed in mitochondria; instead the F_o_F_1_-ATP synthase works in reverse mode such that catalytic rotation of the F_1_ moiety via ATP hydrolysis actively pumps protons out of the mitochondrial matrix via the membrane multimeric F_o_ c-ring pore. The effect of TbFUT1 depletion in insect stage PCF appears to be different. Here, cell death upon TbFUT1 conditional depletion takes longer to manifest than in BSF cells [78]. However, the PCF form of *T. brucei* uses F_o_F_1_-ATP more conventionally, generating ATP from the mitochondrial proton gradient, in conjunction with fully expressed and functional respiratory chain composed of complexes I–IV [81]. Thus, the differences in the kinetics of cell death upon TbFUT1 depletion in the two lifecycle stages may be dependent on their different mitochondrial bioenergetics and/or protein expression requirements. Furthermore, an *LmFUT1*-null segregant mutant (*Δfut1^s^*) exhibits impaired mitochondrial function, including a reduced mitochondrial membrane potential, abnormalities in mitochondrial structure and impaired biosynthesis of the kinetoplast DNA network [79]. Together, these findings implicate a general requirement of FUT1 for normal mitochondrial function in kinetoplastids. Work is ongoing to tease apart the functions of TbFUT1 in BSF and PCF *T. brucei* and *LmFUT1* in *L. major*.

### Putative mitochondrial GT25 family enzymes

Since LNB is a substrate for recombinant TbFUT1 [78], we postulate that there may be other GTs that assemble Galβ1–3GlcNAc on mitochondrial acceptor molecules (protein, lipid or other) on which TbFUT1 can act. These putative preceding GTs could be cytosolic, such that Galβ1–3GlcNAc primed molecules could be imported into the mitochondrion for fucosylation, or they could be mitochondrial themselves. Significantly, a GT25 family enzyme (TbGTX) is predicted to be mitochondrial by mitochondrial import analysis [82] and both TbGTX and another GT25 family member (TbGTZ) have been localised to the mitochondrion in *T. brucei* by C-terminal GFP tagging and fluorescence microscopy [83]. Our own C-terminal epitope tagging studies also localise TbGTX and TbGTZ to the mitochondrion and RNAi studies in BSF cells show that, like TbFUT1, they are both essential (unpublished data). We also find proteomic evidence for the expression of TbGTX in both BSF and PCF cells (Figure 3). Thus, we hypothesise that TbGTX, TbGTZ and TbFUT1 may be involved in the biosynthesis of Fucα1–2Galβ1–3GlcNAc trisaccharide motifs attached to mitochondrial molecules in *T. brucei*. Further studies are required to test this.

Phylogenetic analysis indicates that syntenic orthologues of TbGTX and TbGTZ occur in most kintoplastids, with the exception that GTZ is absent from the ancestral, free-living and bacteriophagic kinetoplastid *Bodo saltans* [84]. This might suggest that the GTX sequence has undergone duplication and evolution in the parasitic kinetoplastids. Given the similarity of TbGTX and TbGTZ to bacterial GT25 family members, we suggest that they may have been inherited in a similar manner to TbFUT1, yielding enzymes with conserved function but unique localisation.

## Base J glucosyltransferase

Kinetoplastids, uniquely, contain small amounts of β-d-glucopyranosyloxymethyluracil (base J) in their DNA. The synthesis of base J involves the formation of 5-hydroxymethyluracil (hmU) and its subsequent glucosylation. The nuclear-localised, base J-specific GT (JGT) [85] has a GT-A fold; however, it is not currently assigned to any CAZy family. Base J does not appear to be essential for trypanosome survival [85], but it appears to play a regulatory role in Pol II-mediated polycistronic gene regulation [86].

## Functions looking for GTs and GTs looking for functions in *T. brucei*

### UDP-sugar: polypeptide GTs

In *T. cruzi*, a small gene family (*TcOGNT1*, *TcOGNT2* and *TcOGNTL*), belonging to the CAZy GT60 family, has been described [87,88]. TcOGNT1 has been shown to be a Golgi located UDP-GlcNAc : polypepetide αGlcNAc-transferase, making GlcNAcα1-*O*-Thr linkages in the abundant surface GPI-anchored mucin-like molecules of this parasite [87,88]. In these mucin-like glycoproteins, the *O*-linked GlcNAc residue can be variously substituted with βGal and β-galactofuranose (βGal*f*) residues, where terminal βGal residues can be capped with α2–3 linked sialic acid via trans-sialidase activity [89]. However, no such mucin-like molecules or similar *O*-linked glycans have been found in *T. brucei*, begging the question as to what the GT60 *TbOGNT1* gene product, which is lowly expressed in BSF and PCF cells (Figure 3), might be doing in this organism. One possibility worth exploring is whether it might be involved in the formation of the novel Glcα1-*O*-Ser linkage observed in several *T. brucei* VSGs [30], for which no GT gene has been assigned thus far. Similarly, the TbGT(s) responsible for adding up to two more hexoses to Glcα1-*O*-Ser remain to be identified.

### α-Galactosyltransferases

Since GTs within a given CAZy GT family generally encode either inverting (β) or retaining (α) GTs, and since all GT67 family members thus far have proven to be β-glycosyltransferases, we most likely need to look outside of the GT67 group for BSF-specific αGal-transferases. Such αGal-transferases must exist to cap small complex *N*-glycans with Galα1–3Gal motifs, as found in some VSGs [90], and for the αGal-transferases that decorate the BSF GPI anchors [24,91] (Figure 1). Searches for the former using CAZy GT6, GT8 and GT77 family sequences fail to return convincing hits. There are no precedents for αGal-transferases that decorate GPI anchors, nor do the trypanosome genomes contain anything like PGAP4 that encodes a mammalian Golgi UDP-GalNAc : GPI β1–4GalNAc-transferase [92]. Although a putative UDP-Gal : GPI αGaT activity was previously partially purified from *T. brucei* whole cell lysates [93], the protein and gene sequences were not identified. Thus, all of the trypanosome αGalT genes remain to be identified and may constitute new GT families.

Summary

In this review, we have discussed

- The inversion of glycosylation complexity between the BSF cells (simple GPI sidechains and complex *N*-glycans) and PCF cells (complex GPI sidechains and simple *N*-glycans) (Figure 1).
- How the selection of complex and/or simple *N*-glycans is made through the expression TbSTT3A and/or TbSTT3B OSTs, and how ER quality control has been adapted in *T. brucei* to cope with its prodigious flux of surface VSG molecules.
- The acquisition of an ancestral eukaryotic β3GT gene and its expansion to create the kinetoplastid-specific GT67 family that has acquired numerous UDP-Gal/GlcNAc βGal/GlcNAc-transferase functions, providing a clear example of GT convergent evolution.
- The speculation that certain GT67 sub-families may encode the elusive UDP-Gal : βGlcNAc β1–4 Gal-transferases required for poly-LacNAc synthesis in BSF and PCF *T. brucei*.
- The identification of essential mitochondrial GTs of bacterial origin in the kinetoplastids, with some speculation as to their function.
- The existence of a glucosyltransferase to make the kinetoplastid-specific modified DNA nucleotide base J.
- The *T. brucei* GT67, GT25 and GT60 family TbGTs still looking for a function, and glycosidic linkages in *T. brucei* still looking for the TbGTs that catalyse them (including several αGal-transferases).

These elements indicate the progress that has been made in understanding protein glycosylation and glycosylation machinery in trypanosomes in recent years. Progress that has both unearthed potential drug targets and led to the discovery of novel biology in *T. brucei* and its pathogenic relatives.

## Open Access

Open access for this article was enabled by the participation of the University of Dundee in an all-inclusive *Read & Publish* agreement with Portland Press and the Biochemical Society under a transformative agreement with JISC.

## Abbreviations

BSF: bloodstream form
CAZy: Carbohydrate Enzyme
ER: endoplasmic reticulum
FUT: fucosyltransferase
GARPs: glutamic acid and alanine-rich glycoproteins
GPI: glycosylphosphatidylinositol
GT: glycosyltransferases
HAT: human African trypanosomiasis
LLO: lipid-linked oligosaccharide
OST: oligosaccharyltransferase
PCF: procyclic form
VSG: variant surface glycoprotein

## References

[1] Shaw, A.P.M., Cecchi, G., Wint, G.R.W., Mattioli, R.C. and Robinson, T.P. (2014) Mapping the economic benefits to livestock keepers from intervening against bovine trypanosomosis in Eastern Africa. Prev. Vet. Med. 113, 197–210 10.1016/j.prevetmed.2013.10.024 24275205

[2] Shrimal, S. and Gilmore, R. (2019) Oligosaccharyltransferase structures provide novel insight into the mechanism of asparagine-linked glycosylation in prokaryotic and eukaryotic cells. Glycobiology 29, 288–297 10.1093/glycob/cwy093 30312397 PMC6499010

[3] Stanley, P., Moremen, K.W., Lewis, N.E., Taniguchi, N. and Aebi, M. (2022) N-Glycans. In Essentials of Glycobiology (Varki, A., Cummings, R.D., Esko, J.D., Stanley, P., Hart, G.W., Aebi, M. et al. eds), pp. 103–116, Cold Spring Harbor, NY

[4] Komath, S.S., Fujita, M., Hart, G.W., Ferguson, M.A.J. and Kinoshita, T. (2022) Glycosylphosphatidylinositol anchors. In Essentials of Glycobiology (Varki, A., Cummings, R.D., Esko, J.D., Stanley, P., Hart, G.W., Aebi, M. et al. eds), pp. 141–154, Cold Spring Harbor, NY

[5] Albuquerque-Wendt, A., Hütte, H.J., Buettner, F.F.R., Routier, F.H. and Bakker, H. (2019) Membrane topological model of glycosyltransferases of the GT-C superfamily. Int. J. Mol. Sci. 20, 1–16 10.3390/ijms20194842 PMC6801728 31569500

[6] Samuelson, J., Banerjee, S., Magnelli, P., Cui, J., Kelleher, D.J., Gilmore, R. et al. (2005) The diversity of dolichol-linked precursors to Asn-linked glycans likely results from secondary loss of sets glycosyltranferases. Proc. Natl Acad. Sci. U.S.A. 102, 1548–1553 10.1073/pnas.0409460102 15665075 PMC545090

[7] Leal, S., Acosta-Serrano, A., Morris, J. and Cross, G.A.M. (2004) Transposon mutagenesis of *Trypanosoma brucei* identifies glycosylation mutants resistant to concanavalin A. J. Biol. Chem. 279, 28979–28988 10.1074/jbc.M403479200 15123607

[8] Manthri, S., Güther, M.L.S., Izquierdo, L., Acosta-Serrano, A. and Ferguson, M.A.J. (2008) Deletion of the TbALG3 gene demonstrates site-specific *N*-glycosylation and *N*-glycan processing in *Trypanosoma brucei*. Glycobiology 18, 367–383 10.1093/glycob/cwn014 18263655

[9] Nagamune, K., Nozaki, T., Maeda, Y., Ohishi, K., Fukuma, T., Hara, T., et al. (2000) Critical roles of glycosylphosphatidylinositol for *Trypanosoma brucei*. Proc. Natl Acad. Sci.U.S.A. 97, 10336–10341 10.1073/pnas.180230697 10954751 PMC27025

[10] Izquierdo, L., Mehlert, A. and Ferguson, M.A. (2012) The lipid-linked oligosaccharide donor specificities of *Trypanosoma brucei* oligosaccharyltransferases. Glycobiology 22, 696–703 10.1093/glycob/cws003 22241825 PMC3311286

[11] Lairson, L.L., Henrissat, B., Davies, G.J. and Withers, S.G. (2008) Glycosyltransferases: structures, functions, and mechanisms. Annu. Rev. Biochem. 77, 521–555 10.1146/annurev.biochem.76.061005.092322 18518825

[12] Taujale, R., Zhou, Z., Yeung, W., Moremen, K.W., Li, S. and Kannan, N. (2021) Mapping the glycosyltransferase fold landscape using interpretable deep learning. Nat. Commun. 12, 5656 10.1038/s41467-021-25975-9 34580305 PMC8476585

[13] Cantarel, B.L., Coutinho, P.M., Rancurel, C., Bernard, T., Lombard, V. and Henrissat, B. (2009) The carbohydrate-active enZymes database (CAZy): an expert resource for glycogenomics. Nucleic Acids Res. 37, 233–238 10.1093/nar/gkn663 PMC2686590 18838391

[14] Taujale, R., Venkat, A., Huang, L.C., Zhou, Z., Yeung, W., Rasheed, K.M. et al. (2020) Deep evolutionary analysis reveals the design principles of fold a glycosyltransferases. eLife 9, 1–24 10.7554/eLife.54532 PMC7185993 32234211

[15] Izquierdo, L., Nakanishi, M., Mehlert, A., Machray, G., Barton, G.J. and Ferguson, M.A.J. (2009) Identification of a glycosylphosphatidylinositol anchor-modifying β1-3 *N*-acetylglucosaminyl transferase in *Trypanosoma brucei*. Mol. Microbiol. 71, 478–491 10.1111/j.1365-2958.2008.06542.x 19040631

[16] Damerow, M., Graalfs, F., Güther, M.L.S., Mehlert, A., Izquierdo, L. and Ferguson, M.A.J. (2016) A gene of the β3-glycosyltransferase family encodes *N*-acetylglucosaminyltransferase II function in *Trypanosoma brucei*. J. Biol. Chem. 291, 13834–13845 10.1074/jbc.M116.733246 27189951 PMC4919465

[17] Damerow, M., Rodrigues, J.A., Wu, D., Güther, M.L.S., Mehlert, A. and Ferguson, M.A.J. (2014) Identification and functional characterization of a highly divergent *N*-acetylglucosaminyltransferase I (TbGnTI) in *Trypanosoma brucei*. J. Biol. Chem. 289, 9328–9339 10.1074/jbc.M114.555029 24550396 PMC3979372

[18] Duncan, S.M., Nagar, R., Damerow, M., Yashunsky, D.V., Buzzi, B., Nikolaev, A.V. et al. (2021) A *Trypanosoma brucei* β3 glycosyltransferase superfamily gene encodes a β1-6 GlcNAc-transferase mediating *N*-glycan and GPI anchor modification. J. Biol. Chem. 297, 14–16 10.1016/j.jbc.2021.101153 PMC8477195 34478712

[19] Izquierdo, L., Acosta-Serrano, A., Mehlert, A. and Ferguson, M.A. (2015) Identification of a glycosylphosphatidylinositol anchor-modifying β1-3 galactosyltransferase in *Trypanosoma brucei*. Glycobiology 25, 438–447 10.1093/glycob/cwu131 25467966 PMC4339879

[20] Nakanishi, M., Karasudani, M., Shiraishi, T., Hashida, K., Hino, M., Ferguson, M.A. et al. (2014) TbGT8 is a bifunctional glycosyltransferase that elaborates N-linked glycans on a protein phosphatase AcP115 and a GPI-anchor modifying glycan in *Trypanosoma brucei*. Parasitol. Int. 63, 513–518 10.1016/j.parint.2014.01.007 24508870 PMC4003530

[21] Capewell, P., Cren-Travaillé, C., Marchesi, F., Johnston, P., Clucas, C., Benson, R.A., et al. (2016) The skin is a significant but overlooked anatomical reservoir for vector-borne African trypanosomes. eLife 5, e17716 10.7554/eLife.17716 27653219 PMC5065312

[22] Trindade, S., Rijo-Ferreira, F., Carvalho, T., Pinto-Neves, D., Guegan, F., Aresta-Branco, F., et al. (2016) *Trypanosoma brucei* parasites occupy and functionally adapt to the adipose tissue in mice. Cell Host Microbe 19, 837–848 10.1016/j.chom.2016.05.002 27237364 PMC4906371

[23] Bartossek, T., Jones, N.G., Schäfer, C., Cvitković, M., Glogger, M., Mott, H.R., et al. (2017) Structural basis for the shielding function of the dynamic trypanosome variant surface glycoprotein coat. Nat. Microbiol. 2, 1523–1532 10.1038/s41564-017-0013-6 28894098

[24] Mehlert, A., Richardson, J.M. and Ferguson, M.A.J. (1998) Structure of the glycosylphosphatidylinositol membrane anchor glycan of a class-2 variant surface glycoprotein from *Trypanosoma brucei*. J. Mol. Biol. 277, 379–392 10.1006/jmbi.1997.1600 9514751

[25] Mehlert, A., Bond, C.S. and Ferguson, M.A.J.J. (2002) The glycoforms of a *Trypanosoma brucei* variant surface glycoprotein and molecular modeling of a glycosylated surface coat. Glycobiology 12, 607–612 10.1093/glycob/cwf079 12244073

[26] Schwede, A., Macleod, O.J.S., MacGregor, P. and Carrington, M. (2015) How does the VSG coat of bloodstream form african trypanosomes interact with external proteins? PLoS Pathog. 11, 1–18 10.1371/journal.ppat.1005259 PMC4697842 26719972

[27] Horn, D. (2014) Antigenic variation in African trypanosomes. Mol. Biochem. Parasitol. 195, 123–129 10.1016/j.molbiopara.2014.05.001 24859277 PMC4155160

[28] Mehlert, A. (1998) The glycosylation of the variant surface glycoproteins and procyclic acidic repetitive proteins of *Trypanosoma brucei*. Mol. Biochem. Parasitol. 91, 145–152 10.1016/S0166-6851(97)00187-4 9574932

[29] Hartel, A.J.W., Glogger, M., Jones, N.G., Abuillan, W., Batram, C., Hermann, A. et al. (2016) *N*-glycosylation enables high lateral mobility of GPI-anchored proteins at a molecular crowding threshold. Nat. Commun. 7, 12870 10.1038/ncomms12870 27641538 PMC5031801

[30] Pinger, J., Nešić, D., Ali, L., Aresta-Branco, F., Lilic, M., Chowdhury, S., et al. (2018) African trypanosomes evade immune clearance by *O*-glycosylation of the VSG surface coat. Nat. Microbiol. 3, 932–938 10.1038/s41564-018-0187-6 29988048 PMC6108419

[31] Mehlert, A. and Ferguson, M.A.J. (2007) Structure of the glycosylphosphatidylinositol anchor of the *Trypanosoma brucei* transferrin receptor. Mol. Biochem. Parasitol. 151, 220–223 10.1016/j.molbiopara.2006.11.001 17140675

[32] Mehlert, A., Wormald, M.R. and Ferguson, M.A.J. (2012) Modeling of the *N*-glycosylated transferrin receptor suggests how transferrin binding can occur within the surface coat of *Trypanosoma brucei*. PLoS Pathog. 8, 1–11 10.1371/journal.ppat.1002618 PMC3320590 22496646

[33] Koeller, C.M., Tiengwe, C., Schwartz, K.J. and Bangs, J.D. (2020) Steric constraints control processing of glycosylphosphatidylinositol anchors in *Trypanosoma brucei*. J. Biol. Chem. 295, 2227–2238 10.1074/jbc.RA119.010847 31932305 PMC7039559

[34] Atrih, A., Richardson, J.M., Prescott, A.R. and Ferguson, M.A.J. (2005) *Trypanosoma brucei* glycoproteins contain novel giant poly-*N*-acetyllactosamine carbohydrate chains. J. Biol. Chem. 280, 865–871 10.1074/jbc.M411061200 15509560

[35] Nolan, D.P., Geuskens, M. and Pays, E. (1999) N-linked glycans containing linear poly-*N*-acetyllactosamine as sorting signals in endocytosis in *Trypanosoma brucei*. Curr. Biol. 9, 1169–1172 10.1016/S0960-9822(00)80018-4 10531030

[36] Güther, M.L.S., Beattie, K., Lamont, D.J., James, J., Prescott, A.R. and Ferguson, M.A.J. (2009) Fate of glycosylphosphatidylinositol (GPI)-less procyclin and characterization of sialylated non-GPI-anchored surface coat molecules of procyclic-form *Trypanosoma brucei*. Eukaryot. Cell 8, 1407–1417 10.1128/EC.00178-09 19633269 PMC2747833

[37] Roditi, I., Schwarz, H., Pearson, T.W., Beecroft, R.P., Liu, M.K., Richardson, J.P., et al. (1989) Procyclin gene expression and loss of the variant surface glycoprotein during differentiation of *Trypanosoma brucei*. J. Cell Biol. 108, 737–746 10.1083/jcb.108.2.737 2645304 PMC2115453

[38] Vassella, E., Bütikofer, P., Engstler, M., Jelk, J. and Roditi, I. (2003) Procyclin null mutants of *Trypanosoma brucei* express free glycosylphosphatidylinositols on their surface. Mol. Biol. Cell 14, 1308–1318 10.1091/mbc.e02-10-0694 12686589 PMC153102

[39] Güther, M.L.S., Lee, S., Tetley, L., Acosta-Serrano, A. and Ferguson, M.A.J. (2006) GPI-anchored proteins and free GPI glycolipids of procyclic form *Trypanosoma brucei* are nonessential for growth, are required for colonization of the tsetse fly, and are not the only components of the surface coat. Mol. Biol. Cell 17, 5265–5274 10.1091/mbc.e06-08-0702 17035628 PMC1679689

[40] Treumann, A., Zitzmann, N., Hülsmeier, A., Prescott, A.R., Almond, A., Sheehan, J. et al. (1997) Structural characterisation of two forms of procyclic acidic repetitive protein expressed by procyclic forms of *Trypanosoma brucei*. J. Mol. Biol. 269, 529–547 10.1006/jmbi.1997.1066 9217258

[41] Mehlert, A., Treumann, A. and Ferguson, M.A.J. (1999) *Trypanosoma brucei* GPEET-PARP is phosphorylated on six out of seven threonine residues. Mol. Biochem. Parasitol. 98, 291–296 10.1016/S0166-6851(98)00168-6 10080398

[42] Ferguson, M.A., Murray, P., Rutherford, H. and McConville, M.J. (1993) A simple purification of procyclic acidic repetitive protein and demonstration of a sialylated glycosyl-phosphatidylinositol membrane anchor. Biochem. J. 291, 51–55 10.1042/bj2910051 8471053 PMC1132479

[43] Engstler, M., Reuter, G. and Schauer, R. (1993) The developmentally regulated trans-sialidase from *Trypanosoma brucei* sialylates the procyclic acidic repetitive protein. Mol. Biochem. Parasitol. 61, 1–13 10.1016/0166-6851(93)90153-O 8259122

[44] Nagamune, K., Acosta-Serrano, A., Uemura, H., Brun, R., Kunz-Renggli, C., Maeda, Y. et al. (2004) Surface sialic acids taken from the host allow trypanosome survival in tsetse fly vectors. J. Exp. Med. 199, 1445–1450 10.1084/jem.20030635 15136592 PMC2211819

[45] Acosta-Serrano, A., Vassella, E., Liniger, M., Kunz Renggli, C., Brun, R., Roditi, I. et al. (2001) The surface coat of procyclic *Trypanosoma brucei*: programmed expression and proteolytic cleavage of procyclin in the tsetse fly. Proc. Natl Acad. Sci. U.S.A. 98, 1513–1518 10.1073/pnas.98.4.1513 11171982 PMC29288

[46] Thomson, L.M., Lamont, D.J., Mehlert, A., Barry, J.D. and Ferguson, M.A.J. (2002) Partial structure of glutamic acid and alanine-rich protein, a major surface glycoprotein of the insect stages of *Trypanosoma congolense*. J. Biol. Chem. 277, 48899–48904 10.1074/jbc.M208942200 12368279

[47] Nolan, D.P., Jackson, D.G., Biggs, M.J., Brabazon, E.D., Pays, A., Van Laethem, F. et al. (2000) Characterization of a novel alanine-rich protein located in surface microdomains in *Trypanosoma brucei*. J. Biol. Chem. 275, 4072–4080 10.1074/jbc.275.6.4072 10660566

[48] Urwyler, S., Studer, E., Renggli, C.K. and Roditi, I. (2007) A family of stage-specific alanine-rich proteins on the surface of epimastigote forms of *Trypanosoma brucei*. Mol. Microbiol. 63, 218–228 10.1111/j.1365-2958.2006.05492.x 17229212

[49] Casas-Sánchez, A., Perally, S., Ramaswamy, R., Haines, L.R., Rose, C., Yunta, C., et al. (2018) The crystal structure and localization of *Trypanosoma brucei* invariant surface glycoproteins suggest a more permissive VSG coat in the tsetse-transmitted metacyclic stage. bioRxiv 477737

[50] Aebi, M. (2013) N-linked protein glycosylation in the ER. Mol. Cell Res. 1833, 2430–2437 10.1016/j.bbamcr.2013.04.001 23583305

[51] Stanley, P. (2011) Golgi glycosylation. Perspect. Biol. 3, a005199 10.1101/cshperspect.a005199 PMC3062213 21441588

[52] Bangs, J.D., Doering, T.L., Englund, P.T. and Hart, G.W. (1988) Biosynthesis of a variant surface glycoprotein of *Trypanosoma brucei*. J. Biol. Chem. 263, 17697–17705 10.1016/S0021-9258(19)77893-4 3182868

[53] Jinnelov, A., Ali, L., Tinti, M., Güther, M.L.S. and Ferguson, M.A.J. (2017) Single-subunit oligosaccharyltransferases of *Trypanosoma brucei* display different and predictable peptide acceptor specificities. J. Biol. Chem. 292, 20328–20341 10.1074/jbc.M117.810945 28928222 PMC5724017

[54] Acosta-Serrano, A., O'Rear, J., Quellhorst, G., Lee, S.H., Hwa, K.-Y., Krag, S.S. et al. (2004) Defects in the *N*-linked oligosaccharide biosynthetic pathway in a *Trypanosoma brucei* glycosylation mutant. Eukaryot. Cell 3, 255–263 10.1128/EC.3.2.255-263.2004 15075256 PMC387663

[55] Jones, D.C. (2005) Deletion of the glucosidase II gene in *Trypanosoma brucei* reveals novel N-glycosylation mechanisms in the biosynthesis of variant surface glycoprotein. J. Biol. Chem. 280, 35929–35942 10.1074/jbc.M509130200 16120601

[56] Izquierdo, L., Schulz, B.L., Rodrigues, J.A., Güther, M.L.S., Procter, J.B., Barton, G.J. et al. (2009) Distinct donor and acceptor specificities of *Trypanosoma brucei* oligosaccharyltransferases. EMBO J. 28, 2650–2661 10.1038/emboj.2009.203 19629045 PMC2722254

[57] Poljak, K., Breitling, J., Gauss, R., Rugarabamu, G., Pellanda, M. and Aebi, M. (2017) Analysis of substrate specificity of *Trypanosoma brucei* oligosaccharyltransferases (OSTs) by functional expression of domain-swapped chimeras in yeast. J. Biol. Chem. 292, 20342–20352 10.1074/jbc.M117.811133 29042445 PMC5724018

[58] Roversi, P., Marti, L., Caputo, A.T., Alonzi, D.S., Hill, J.C., Dent, K.C., et al. (2017) Interdomain conformational flexibility underpins the activity of UGGT, the eukaryotic glycoprotein secretion checkpoint. Proc. Natl Acad. Sci. U.S.A. 114, 8544–8549 10.1073/pnas.1703682114 28739903 PMC5559018

[59] Izquierdo, L., Atrih, A., Rodrigues, J.A., Jones, D.C. and Ferguson, M.A.J. (2009) *Trypanosoma brucei* UDP-glucose:glycoprotein glucosyltransferase has unusual substrate specificity and protects the parasite from stress. Eukaryot. Cell 8, 230–240 10.1128/EC.00361-08 19114500 PMC2643610

[60] Tiengwe, C., Brown, A.E.N.A. and Bangs, J.D. (2015) Unfolded protein response pathways in bloodstream-form *Trypanosoma brucei*? Eukaryot. Cell 14, 1094–1101 10.1128/EC.00118-15 26318397 PMC4621318

[61] Conte, I., Labriola, C., Cazzulo, J.J., Docampo, R. and Parodi, A.J. (2003) The interplay between folding-facilitating mechanisms in *Trypanosoma cruzi* endoplasmic reticulum. Mol. Biol. Cell 14, 3529–3540 10.1091/mbc.e03-04-0228 12972544 PMC196547

[62] Castillo-Acosta, V.M., Vidal, A.E., Ruiz-Pérez, L.M., Van Damme, E.J.M., Igarashi, Y., Balzarini, J. et al. (2013) Carbohydrate-binding agents act as potent trypanocidals that elicit modifications in VSG glycosylation and reduced virulence in *Trypanosoma brucei*. Mol. Microbiol. 90, 665–679 10.1111/mmi.12359 23926900

[63] Castillo-Acosta, V.M., Ruiz-Pérez, L.M., Van Damme, E.J.M., Balzarini, J. and González-Pacanowska, D. (2015) Exposure of *Trypanosoma brucei* to an N-acetylglucosamine-binding lectin induces VSG switching and glycosylation defects resulting in reduced infectivity. PLoS Negl. Trop. Dis. 9, e0003612 10.1371/journal.pntd.0003612 25746926 PMC4351956

[64] Castillo-Acosta, V.M., Ruiz-Pérez, L.M., Etxebarria, J., Reichardt, N.C., Navarro, M., Igarashi, Y. et al. (2016) Carbohydrate-binding non-peptidic pradimicins for the treatment of acute sleeping sickness in murine models. PLOS Pathog. 12, e1005851 10.1371/journal.ppat.1005851 27662652 PMC5035034

[65] Azevedo, L.G., De Queiroz, A.T.L., Barral, A., Santos, L.A. and Ramos, P.I.P. (2020) Proteins involved in the biosynthesis of lipophosphoglycan in *Leishmania*: a comparative genomic and evolutionary analysis. Parasites Vectors 13, 1–14 10.1186/s13071-020-3914-9 32000835 PMC6993435

[66] Pereira, S., and Jackson, S. and P, A. (2018) UDP-glycosyltransferase genes in trypanosomatid genomes have diversified independently to meet the distinct developmental needs of parasite adaptations. BMC Evol. Biol. 18, 31 10.1186/s12862-018-1149-6 29540192 PMC5853035

[67] Cristodero, M., Seebeck, T. and Schneider, A. (2010) Mitochondrial translation is essential in bloodstream forms of *Trypanosoma brucei*. Mol. Microbiol. 78, 757–769 10.1111/j.1365-2958.2010.07368.x 20969649

[68] Tinti, M., Güther, M.L.S., Crozier, T.W.M., Lamond, A.I. and Ferguson, M.A.J. (2019) Proteome turnover in the bloodstream and procyclic forms of *Trypanosoma brucei* measured by quantitative proteomics. Wellcome Open Res. 4, 152 10.12688/wellcomeopenres.15421.1 31681858 PMC6816455

[69] Ferguson, M.A.J. and Tinti, M. (2022) Visualisation of proteome-wide ordered protein abundances in *Trypanosoma brucei*. Wellcome Open Res. 7, 1–11 10.12688/wellcomeopenres.17428.2 35284642 PMC8889043

[70] Gardiner, P.R., Nene, V., Barry, M.M., Thatthi, R., Burleigh, B. and Clarke, M.W. (1996) Characterization of a small variable surface glycoprotein from *Trypanosoma vivax*. Mol. Biochem. Parasitol. 82, 1–11 10.1016/0166-6851(96)02687-4 8943146

[71] Bendiak, B. and Schachter, H. (1987) Control of glycoprotein synthesis. Kinetic mechanism, substrate specificity, and inhibition characteristics of UDP-*N*-acetylglucosamine:alpha-D-mannoside beta 1-2 *N*-acetylglucosaminyltransferase II from rat liver. J. Biol. Chem. 262, 5784–5790 10.1016/S0021-9258(18)45643-8 2952645

[72] Wang, Y., Tan, J., Sutton-Smith, M., Ditto, D., Panico, M., Campbell, R.M., et al. (2001) Modelling human congenital disorder of glycosylation type IIa in the mouse: conservation of asparagine-linked glycan-dependent functions in mammalian physiology and insights into disease pathogenesis. Glycobiology 11, 1051–1070 10.1093/glycob/11.12.1051 11805078

[73] Ioffe, E. and Stanley, P. (1993) Mice lacking *N*-acetylglucosaminyltransferase I activity die at mid-gestation, revealing an essential role for complex or hybrid *N*-linked carbohydrates. Proc. Natl Acad. Sci. U.S.A. 91, 728–732 10.1073/pnas.91.2.728 PMC43022 8290590

[74] Jinelov, P. (2014) The Investigation of Oligosaccharyltransferase in *Trypanosoma brucei*. PhD Thesis, University of Dundee, pp. 59–78

[75] Peck, R.F., Shiflett, A.M., Schwartz, K.J., McCann, A., Hajduk, S.L. and Bangs, J.D. (2008) The LAMP-like protein p67 plays an essential role in the lysosome of African trypanosomes. Mol. Microbiol. 68, 933–946 10.1111/j.1365-2958.2008.06195.x 18430083

[76] Turnock, D.C., Izquierdo, L. and Ferguson, M.A.J. (2007) The *de novo* synthesis of GDP-fucose is essential for flagellar adhesion and cell growth in *Trypanosoma brucei*. J. Biol. Chem. 282, 28853–28863 10.1074/jbc.M704742200 17640865

[77] Guo, H., Novozhilova, N.M., Bandini, G., Turco, S.J., Ferguson, M.A.J. and Beverley, S.M. (2017) Genetic metabolic complementation establishes a requirement for GDP-fucose in *Leishmania*. J. Biol. Chem. 292, 10696–10708 10.1074/jbc.M117.778480 28465349 PMC5481574

[78] Bandini, G., Damerow, S., Sempaio Guther, M.L., Guo, H., Mehlert, A., Paredes Franco, J.C. et al. (2021) An essential, kinetoplastid-specific GDP-Fuc: β-D-Gal α-1,2-fucosyltransferase is located in the mitochondrion of *Trypanosoma brucei*. eLife 10, 1–27 10.7554/eLife.70272 PMC8439653 34410224

[79] Guo, H., Damerow, S., Penha, L., Menzies, S., Polanco, G., Zegzouti, H. et al. (2021) A broadly active fucosyltransferase LmjFUT1 whose mitochondrial localization and activity are essential in parasitic leishmania. Proc. Natl Acad. Sci. U.S.A. 118, e2108963118 10.1073/pnas.2108963118 34385330 PMC8379939

[80] Irwin, N.A.T., Pittis, A.A., Richards, T.A. and Keeling, P.J. (2022) Systematic evaluation of horizontal gene transfer between eukaryotes and viruses. Nat. Microbiol. 7, 327–336 10.1038/s41564-021-01026-3 34972821

[81] Hierro-Yap, C., Šubrtová, K., Gahura, O., Panicucci, B., Dewar, C., Chinopoulos, C. et al. (2021) Bioenergetic consequences of F_o_F_1_-ATP synthase/ATPase deficiency in two life cycle stages of *Trypanosoma brucei*. J. Biol. Chem. 296, 100357. 10.1016/j.jbc.2021.100357 33539923 PMC7949148

[82] Peikert, C.D., Mani, J., Morgenstern, M., Käser, S., Knapp, B., Wenger, C. et al. (2017) Charting organellar importomes by quantitative mass spectrometry. Nat. Commun. 8, 15272 10.1038/ncomms15272 28485388 PMC5436138

[83] Dean, S., Sunter, J.D. and Wheeler, R.J. (2017) Tryptag.org: a trypanosome genome-wide protein localisation resource. Trends Parasitol. 33, 80–82 10.1016/j.pt.2016.10.009 27863903 PMC5270239

[84] Jackson, A.P., Quail, M.A. and Berriman, M. (2008) Insights into the genome sequence of a free-living kinetoplastid: *Bodo saltans* (Kinetoplastida: Euglenozoa). BMC Genom. 9, 594 10.1186/1471-2164-9-594 PMC2621209 19068121

[85] Bullard, W., Lopes Da Rosa-Spiegler, J., Liu, S., Wang, Y. and Sabatini, R. (2014) Identification of the glucosyltransferase that converts hydroxymethyluracil to base J in the trypanosomatid genome. J. Biol. Chem. 289, 20273–20282 10.1074/jbc.M114.579821 24891501 PMC4106341

[86] Kieft, R., Zhang, Y., Marand, A.P., Moran, J.D., Bridger, R., Wells, L. et al. (2020) Identification of a novel base J binding protein complex involved in RNA polymerase II transcription termination in trypanosomes. PLOS Genet. 16, e1008390 10.1371/journal.pgen.1008390 32084124 PMC7055916

[87] Heise, N., Singh, D., van der Wel, H., Sassi, S.O., Johnson, J.M., Feasley, C.L. et al. (2009) Molecular analysis of a UDP-GlcNAc: polypeptide α-*N*-acetylglucosaminyltransferase implicated in the initiation of mucin-type *O*-glycosylation in *Trypanosoma cruzi*. Glycobiology 19, 918–933 10.1093/glycob/cwp068 19468051 PMC2704902

[88] Koeller, C.M., van der Wel, H., Feasley, C.L., Abreu, F., da Rocha, J.D.B., Montalvão, F., et al. (2014) Golgi UDP-GlcNAc:Polypeptide *O*-α-*N*-Acetyl-D-glucosaminyltransferase 2 (TcOGNT2) regulates trypomastigote production and function in *Trypanosoma cruzi*. Eukaryot. Cell 13, 1312–1327 10.1128/EC.00165-14 25084865 PMC4187647

[89] Jones, C., Todeschini, A.R., Agrellos, O.A., Previato, J.O. and Mendonça-Previato, L. (2004) Heterogeneity in the biosynthesis of mucin *O* -Glycans from *Trypanosoma cruzi* tulahuen strain with the expression of novel galactofuranosyl-containing oligosaccharides. Biochemistry 43, 11889–11897 10.1021/bi048942u 15362875

[90] Zamze, S.E., Ashford, D.A., Wooten, E.W., Rademacher, T.W. and Dwek, R.A. (1991) Structural characterization of the asparagine-linked oligosaccharides from *Trypanosoma brucei* type II and type III variant surface glycoproteins. J. Biol. Chem. 266, 20244–20261 10.1016/S0021-9258(18)54916-4 1939085

[91] Ferguson, M.A.J., Homans, S.W., Dwek, R.A. and Rademacher, T.W. (1988) Glycosyl-phosphatidylinositol moiety that anchors *Trypanosoma brucei* variant surface glycoprotein to the membrane. Science 239, 753–759 10.1126/science.3340856 3340856

[92] Hirata, T., Mishra, S.K., Nakamura, S., Saito, K., Motooka, D., Takada, Y. et al. (2018) Identification of a Golgi GPI-*N*-acetylgalactosamine transferase with tandem transmembrane regions in the catalytic domain. Nat. Commun. 9, 405 10.1038/s41467-017-02799-0 29374258 PMC5785973

[93] Pingel, S., Rheinweiler, U., Kolb, V. and Duszenko, M. (1999) Purification and characterization of an α-galactosyltransferase from *Trypanosoma brucei*. Biochem. J. 338, 545–551 10.1042/bj3380545 10024534 PMC1220084

